# Association between physiological serum total bilirubin concentrations and the progression of diabetic nephropathy

**DOI:** 10.3389/fendo.2025.1588568

**Published:** 2025-05-29

**Authors:** Chenhui Zhang, Yuan Fang, Zishan Lin, Yongjie Zhuo, Jianxin Wan, Xiaohong Zhang

**Affiliations:** ^1^ The First Clinical College of Fujian Medical University, Fuzhou, China; ^2^ Department of Nephrology, Blood Purification Research Center, The First Affiliated Hospital, Fujian Medical University, Fuzhou, China; ^3^ Fujian Clinical Research Center for Metabolic Chronic Kidney Disease, The First Affiliated Hospital, Fujian Medical University, Fuzhou, China; ^4^ Department of Nephrology, National Regional Medical Center, Binhai Campus of the First Affiliated Hospital, Fujian Medical University, Fuzhou, China

**Keywords:** diabetic nephropathy, serum total bilirubin, risk factor, progression, restricted cubic spline

## Abstract

**Purpose:**

To analyze the relationship between physiological serum total bilirubin (STB) concentrations and the progression of diabetic nephropathy (DN).

**Methods:**

The clinical features and pathological data of 159 patients with diabetic nephropathy confirmed by renal biopsy were retrospectively analyzed. They were divided into low bilirubin group (80 cases) and high bilirubin group (79 cases) according to the median of STB level. Clinical and pathological data of the two groups were collected and compared. The patients were followed up from the date of renal biopsy to June 30, 2024. Kaplan-Meier method and log-rank test was used to perform survival analysis. Univariate and multivariate Cox regression risk model were used to analyze the risk factors of diabetic nephropathy. A restricted cubic spline model was used to show the nonlinear association between STB and DN.

**Results:**

When compared with physiologically high bilirubin group, patients in low bilirubin group might had higher level of serum creatinine, blood urea nitrogen, 24h urinary protein, urinary albumin to creatinine ratio (UACR), fibrinogen (Fib) and higher rate of *K-W* nodules, renal tubular atrophy, renal interstitial inflammation and lower level of eGFR, hemoglobin, PLT, suggesting that low bilirubin group had more severe indicators. Spearman correlation analysis showed that STB was positive associated with eGFR (*r* = 0.270, *P < 0.001*) while negative associated with serum BUN (*r* = -0.236, *P* = 0.003), serum creatinine(*r*=-0.256, *P < 0.001*), 24h urine protein(*r* = -0.257, *P < 0.001*), UACR (*r* = -0.287, *P* < 0.001) and Fib (*r* = -0.398, *P* < 0.001). The Kaplan Meier analysis revealed that high STB had a higher possibility of renal survival rate when compared with lower STB (*P* = 0.013). After univariate and multivariate Cox regression analysis, STB (*HR* = 0.445, *P* = 0.001), hemoglobin (*HR* = 0.983, *P* = 0.002), age (*HR* = 0.977,*P* = 0.033) and ACEI/ARB (HR = 0.340, *P* = 0.001) were independently protective factors for the DN progression, while serum creatinine (*HR* = 1.003, *P* =0.001), 24h urine protein (*HR* = 1.088, *P* = 0.005) and cholesterol (*HR* = 1.104, *P* = 0.002) were risk factors for DN progression. The restricted cubic spline model showed that there was a significant nonlinear association between DN progression and STB level when it was less than 6.085 µmol/L

**Conclusions:**

Our findings suggest that STB may serve as a potential biomarker for the progression of diabetic nephropathy. Lower STB levels may help identify high-risk patients who could benefit from earlier or more intensive interventions to slow disease progression.

## 1 Introduction

Diabetic nephropathy (DN) is the leading cause of end-stage renal disease, which causes a heavy healthcare burden worldwide (1), and about 35-40% of patients with type 2 diabetes mellitus (T2DM) develop diabetic nephropathy (2). Epidemiological surveys in China have shown that diabetic nephropathy has surpassed glomerulonephritis as the leading cause of chronic kidney disease in hospitalized patients in China (3). The pathogenesis of diabetic nephropathy is complex and diverse, containing genetics, gene mutations, methylation abnormalities, abnormal hemodynamic fluctuations, inflammatory mediators, oxidative stress, disorders of glucose and lipid metabolism, immune disorders, ischemia and hypoxia, apoptosis and many other factors can be associated with the pathogenesis of DN. But the specific mechanism has not been fully elucidated, in which oxidative stress is closely related to the development of DN (4).

Serum total bilirubin (STB), the end product of catabolism of mammalian hemoglobin, is often considered as waste product, and high levels of bilirubin are potentially neurotoxic. However, bilirubin acts as a potent anti-apoptotic, antioxidant, anti-inflammatory, and immunomodulatory agent at normal or mildly elevated levels, suggesting that bilirubin is cytoprotective. Many recent studies have reported that physiological lower bilirubin levels are associated with the development of diabetic peripheral arterial disease (5), peripheral neuropathy (6, 7), and cardiac autonomic neuropathy (8). A 2017 meta-analysis by Zhu et al. found that a negative nonlinear association between bilirubin concentration and the risk of diabetic complications, such as diabetic nephropathy, diabetic retinopathy and diabetic neuropathy (9).Previous studies have predominantly depended on functional biomarkers, such as estimated glomerular filtration rate (eGFR) and proteinuria. However, these investigations were constrained by the absence of renal biopsy data and a restricted exploration of nonlinear associations. This study aimed to elucidate the relationship between physiological STB concentrations and structural renal damage, as well as to assess its impact on disease progression in patients with diabetic nephropathy.

## 2 Materials and methods

### 2.1 Data source and study population

159 patients who were hospitalized in the Department of Nephrology of the First Affiliated Hospital of Fujian Medical University and diagnosed with diabetic nephropathy after renal biopsy pathology from January 1, 2015 to December 31, 2023 were collected. They were followed up from the date of renal biopsy to June 30, 2024. All patients included in this study were hospitalized primarily due to suspected diabetic nephropathy or the need for comprehensive assessment or adjustment of their diabetes management plan. Biopsy indications almost followed the KDIGO clinical practice guideline for diabetes and CKD:2012 update (10). If a patient has any one of the following conditions, a renal biopsy is recommended: rapid decline in eGFR; sharp increase in proteinuria or sudden-onset nephrotic syndrome; glomerular hematuria; eGFR reduction >30% within 3 months of ACEI/ARB therapy; massive proteinuria without diabetic retinopathy; refractory hypertension; clinical symptoms or signs of systemic diseases. Among them, 108 cases (67.9%) were male and 51 cases (32.1%) were female, aged 53.7 ± 11.3 years. Exclusion criteria: (1) age less than 18 years old; (2) estimated glomerular filtration rate (eGFR) <15 ml/min/(1.73m^2^) at renal biopsy; (3) severe infection; (4) hepatic failure due to various causes; (5) combination of malignant tumors; and (6) patients with incomplete clinical or pathological data and lost visits. All participants included in this study had signed a patient informed consent form upon admission. Throughout the data collection process and afterward, we ensured that no information capable of identifying individual participants was accessed or utilized. The study protocol was reviewed and received approval from the Ethics Review Form for Branch for Medical Research and Clinical Technology Application, Ethics Committee of the First Affiliated Hospital of Fujian Medical University (approval number [2015]084-2).

### 2.2 Clinical and laboratory data

Clinical data were collected at the time of kidney biopsy: Gender, age, BMI (body mass index, kg/m^2^), history of hypertension(yes or no), DM duration(months), cardiovascular disease(yes or no), diabetic retinopathy(yes or no), the use of ACEI/ARB(yes or no), mean arterial pressure (MAP,mmHg),hemoglobin (HB,g/L), platelet(PLT,10^9^/L), serum total bilirubin concentration (STB,umol/L), direct bilirubin concentration (DBIL,µmol/L), indirect bilirubin concentration (IBIL,µmol/L), ALT(U/L), AST(U/L), serum albumin (ALB,g/L), serum creatinine (Scr, umol/L), estimated glomerular filtration rate (eGFR, ml/min/1.73m^2^), blood uric acid (UA, umol/L), serum cholesterol (TC, mmol/L), fibrinogen(Fib, g/L),urine albumin/creatinine (UACR,mg/g), urine protein in 24h (g), HbA1c, CRP (mg/L), TG (mmol/L),HDL-C (mmol/L),LDL-C (mmol/L),Calcium (mmol/L),Phosphorus (mmol/L), Serum C3 (g/L), Serum C4 (g/L) and Serum C1q (g/L) were collected from patients. eGFR was calculated using the Cooperative Chronic Kidney Disease Epidemiology Study (CKD-EPI) formula (11).Longitudinal renal function parameters (e.g., Scr, eGFR) were collected during follow-up to assess disease progression.

### 2.3 Pathological examination

Renal biopsy tissues were examined by light microscopy and immunofluorescence. Glomerular, tubular and interstitial vascular lesions were observed under light microscope, and glomerular spherical sclerosis rate and segmental sclerosis rate (%) were calculated; the points of tubulointerstitial lesions: including the degree of interstitial fibrosis and tubular atrophy (IFTA) and renal interstitial inflammatory cell infiltration, of which the IFTA scoring standard: no lesion is 0 points, small focal lesion (<25%) is 1 point, multifocal lesion (25%∼50%) is 2 points, large sheet-like lesion (50%∼75%) is 3 points, diffuse lesion (>75%) is 4 points; and the scoring criteria for renal interstitial inflammatory cell infiltration: no lesion is 0 points, accompanied by IFTA is 1 point, and visible everywhere is 2 points (12).

### 2.4 Study endpoints

Entry into end stage renal disease (ESRD) starting dialysis.

### 2.5 Statistical methods

The data were processed using R software, version 4.1.2 and SPSS software, version 25.0. For continuous variables, we used the Shapiro-Wilk test to assess whether the data were normally distributed. Data were expressed as mean ± standard deviation if they followed a normal distribution. Comparisons between groups were made using the *t*-test. Data were expressed as medians and interquartile ranges if they were not normally distributed. Count data were expressed as frequencies and percentages, and comparisons between groups of count data were performed using the χ^2^ test. Spearman correlation analysis was used to explore the correlation between serum total bilirubin levels and renal function and pathologic indicators in DN patients. Survival analysis was performed using the Kaplan-Meier method and compared with the Log-rank test, and then the risk factors for renal prognosis in patients with diabetic nephropathy were analyzed using the multifactorial Cox regression model. A restricted cubic spline model was made to show the non-linear relationship between STB and DN. Missing values were input by multiple interpolation for continuous variables. *P* < 0.05 was considered as statistically significant difference.

## 3 Results

### 3.1 Baseline characteristics of participants

A total of 159 participants were enrolled, of whom 67.9% (108) were male, with an average age of 53.7 ± 11.3 years. According to the median of serum total bilirubin level (6.10µmol/L), they were divided into low bilirubin group and high bilirubin group. The clinical and biochemical characteristics of the participants between low bilirubin group and high bilirubin group are shown in **Table 1**. As we can see, in low bilirubin group, patients might had higher level of serum creatinine, blood urea nitrogen, 24h urinary protein, UACR, Fib and higher rate of *K-W* nodules, Renal tubular atrophy, Renal interstitial inflammation and lower level of eGFR, hemoglobin, PLT, suggesting that low bilirubin group had more severe indicators when compared with high bilirubin group.

**Table 1:** Basic characteristics of the participants based on serum total bilirubin level.

Factors	All patients (N=159)	STB level		t/H/χ^2^ value	*P* value
		Low bilirubin group (N=80)	High bilirubin group (N=79)		
General data					
Age (years)	55.00(48.00, 61.00)	55.00(48.00, 61.00)	55.00(47.00, 61.00)	-0.333	0.739
Gender (male,N,%)	108(67.90%)	52(65.00%)	56(70.90%)	0.632	0.427
DM duration (months)	84.00(36.00, 120.00)	96.00(36.00, 144.00)	72.00(24.00, 120.00)	1.373	0.241
BMI (kg/m^2^)	28.83 ± 2.95	23.86 ± 3.16	23.81 ± 2.75	0.114	0.909
MAP (mmHg)	104.68 ± 15.48	103.83 ± 15.67	105.54 ± 16.31	-0.699	0.485
Hypertension (N,%)	141(88.70%)	70(87.50%)	71(89.90%)	0.223	0.637
Cardiovascular disease (N,%)	25(15.70%)	13(16.30%)	12(15.20%)	0.034	0.854
Diabetic retinopathy (N,%)	123(77.40%)	68(85.00%)	55(69.60%)	5.368	0.021^∗^
ACEI/ARB (N,%)	118(74.20%)	57(71.3%)	61(77.2%)	0.739	0.390
Progression (N, %)	90(56.60%)	57(71.30%)	33(41.80%)	14.061	<0.001^∗^
Laboratory data					
STB (µmol/L)	6.10(3.90, 9.30)	3.90(3.20, 4.80)	9.30(7.30, 12.80)	118.530	<0.001^∗^
DBIL (µmol/L)	2.00(1.20, 2.30)	1.35(0.90, 1.98)	2.10(2.00, 3.10)	7.263	<0.001^∗^
IBIL (µmol/L)	4.23(2.80, 5.20)	3.05(1.93, 4.15)	4.90(4.23, 6.30)	7.159	<0.001^∗^
Scr (μmol/L)	132.90(96.00, 176.30)	144.50(109.50, 225.73)	122.10(80.00, 160.00)	7.220	0.007^∗^
eGFR (ml/min/1.73m^2^)	49.79(36.28, 73.28)	44.58(32.95, 62.20)	57.25(40.28, 84.03)	10.090	0.001^∗^
BUN (mmol/L)	9.04(6.50, 14.30)	10.87(7.78, 15.50)	7.58(6.19, 12.38)	8.130	0.004^∗^
Urinary protein in 24h (g/24h)	4.30(2.72, 7.55)	5.39(3.12, 8.95)	3.80(1.60, 5.95)	8.910	0.003^∗^
UACR (mg/g)	3522.41 (1821.96, 5000.00)	4000.00 (2391.58, 5996.20)	2916.53 (1221.91, 4166.26)	12.849	<0.001^∗^
HbA1c (%)	7.70(6.50, 9.10)	7.65(6.50, 9.08)	7.70(6.30, 9.10)	0.000	1.000
Serum albumin (g/L)	29.10(25.60, 34.80)	28.50(24.45, 34.48)	30.60(26.00, 35.30)	0.991	0.319
CRP (mg/L)	5.00(5.00, 6.27)	5.00(3.55, 5.99)	5.00(5.00, 7.01)	0.726	0.394
Hb (g/L)	107.45 ± 22.74	101.95 ± 18.37	113.01 ± 25.36	-3.147	0.002^∗^
PLT (10^9/L)	242.00(198.00, 306.00)	259.50(211.50, 326.75)	235.00(189.00, 280.00)	7.341	0.007^∗^
TC (mmol/L)	5.17(3.85, 6.39)	5.33(1.24, 6.79)	4.87(3.66, 6.27)	1.985	0.159
TG (mmol/L)	1.68(1.12, 2.36)	1.75(1.22, 3.10)	1.62(1.06, 2.09)	2.926	0.087
HDL-C (mmol/L)	1.09(0.89, 1.33)	1.06(0.87, 1.26)	1.11(0.91, 1.40)	1.952	0.162
LDL-C (mmol/L)	3.26(2.29, 4.36)	3.28(2.27, 4.55)	3.26(2.29, 4.32)	0.060	0.807
ALT (U/L)	19.00(13.00, 23.00)	16.00(12.00, 23.00)	21.00(14.00, 23.00)	1.486	0.137
AST (U/L)	20.00(15.00, 24.00)	19.00(14.25, 24.00)	20.00(17.00, 23.00)	1.127	0.260
Calcium (mmol/L)	2.09 ± 0.17	2.09 ± 0.16	2.09 ± 0.18	-0.001	0.999
Phosphorus (mmol/L)	1.28(1.12, 1.45)	1.30(1.12, 1.48)	1.24(1.07, 1.44)	-0.667	0.505
FIB (g/L)	4.55(3.84, 5.55)	5.07(4.38, 6.27)	4.16(3.31, 4.86)	23.261	<0.001^∗^
Uric acid (μmol/L)	383.10(328.00, 434.30)	384.95(319.38, 422.15)	380.10(328.80, 459.80)	0.169	0.681
Urine RBC counts (/HP)	3.62(1.73, 10.32)	4.49(2.05, 9.19)	3.33(1.35, 11.99)	0.967	0.325
Serum C3 (g/L)	0.89(0.80, 1.00)	0.89(0.81, 1.06)	0.87(0.76, 0.94)	4.894	0.027^∗^
Serum C4 (g/L)	0.27(0.21, 0.31)	0.27(0.23, 0.33)	0.25(0.20, 0.28)	-2.617	0.009^∗^
Serum C1q (g/lL)	231.30(223.60, 256.00)	231.30(225.05, 261.23)	231.30(209.70, 250.20)	1.452	0.228
Pathological feature					
Globular glomerulosclerosis rate (%)	22.20(8.30, 39.50)	25.00(9.33, 42.15)	18.20(7.70, 33.30)	1.800	0.180
Segmental glomerulosclerosis rate (%)	4.70(0.00, 31.20)	7.70(0.00, 33.30)	4.20(0.00, 25.00)	0.287	0.592
K-W nodules (N,%)	103(64.80%)	60(75.00%)	43(54.40%)	7.371	0.007^∗^
Renal tubular atrophy(N,%)				11.704	0.008^∗^
0/1	48(30.20%)	15(18.80%)	33(41.80%)		
2	45(28.30%)	23(28.70%)	22(27.80%)		
3	61(38.40%)	39(48.80%)	22(27.80%)		
4	5(3.10%)	3(3.80%)	2(2.50%)		
Renal interstitial inflammation (N,%)				6.844	0.009^∗^
0/1	78(49.10%)	31(38.80%)	47(59.50%)		
2	81(50.90%)	49(61.30%)	32(40.50%)		
Vascular scores (N,%)				5.884	0.117
0	6(3.80%)	3(3.80%)	3(3.80%)		
1	43(27.00%)	15(18.80%)	28(35.40%)		
2	100(62.90%)	57(71.30%)	43(54.40%)		
3	10(6.30%)	5(6.30%)	5(6.30%)		

Data are expressed as means ± standard deviation or medians (interquartile range) or count (%).

^*^ *P* value<0.05.

STB, serum total bilirubin; DM, diabetes mellitus; BMI, body mass index; MAP, mean arterial pressure; ACEI/ARB, angiotensin-converting enzyme inhibitor/angiotensin receptor blockers; DBIL, direct bilirubin; IBIL, indirect bilirubin; HbA1c, glycosylated hemoglobin, type A1c; TC, total cholesterol; TG, triglycerides; HDL-C, high density lipoprotein cholesterol; LDL-C, low density lipoprotein cholesterol; ALT, alanine transaminase; AST, aspartate transaminase; Scr, serum creatinine; eGFR, estimated glomerular filtration rate; CRP, C-reactive protein; Hb, hemoglobin; PLT, platelet; BUN, blood urea nitrogen; UACR, urinary albumin to-creatinine ratio; FIB, fibrinogen; RBC, red blood cell.

### 3.2 The association between STB and renal function indicators

**Figure 1:**
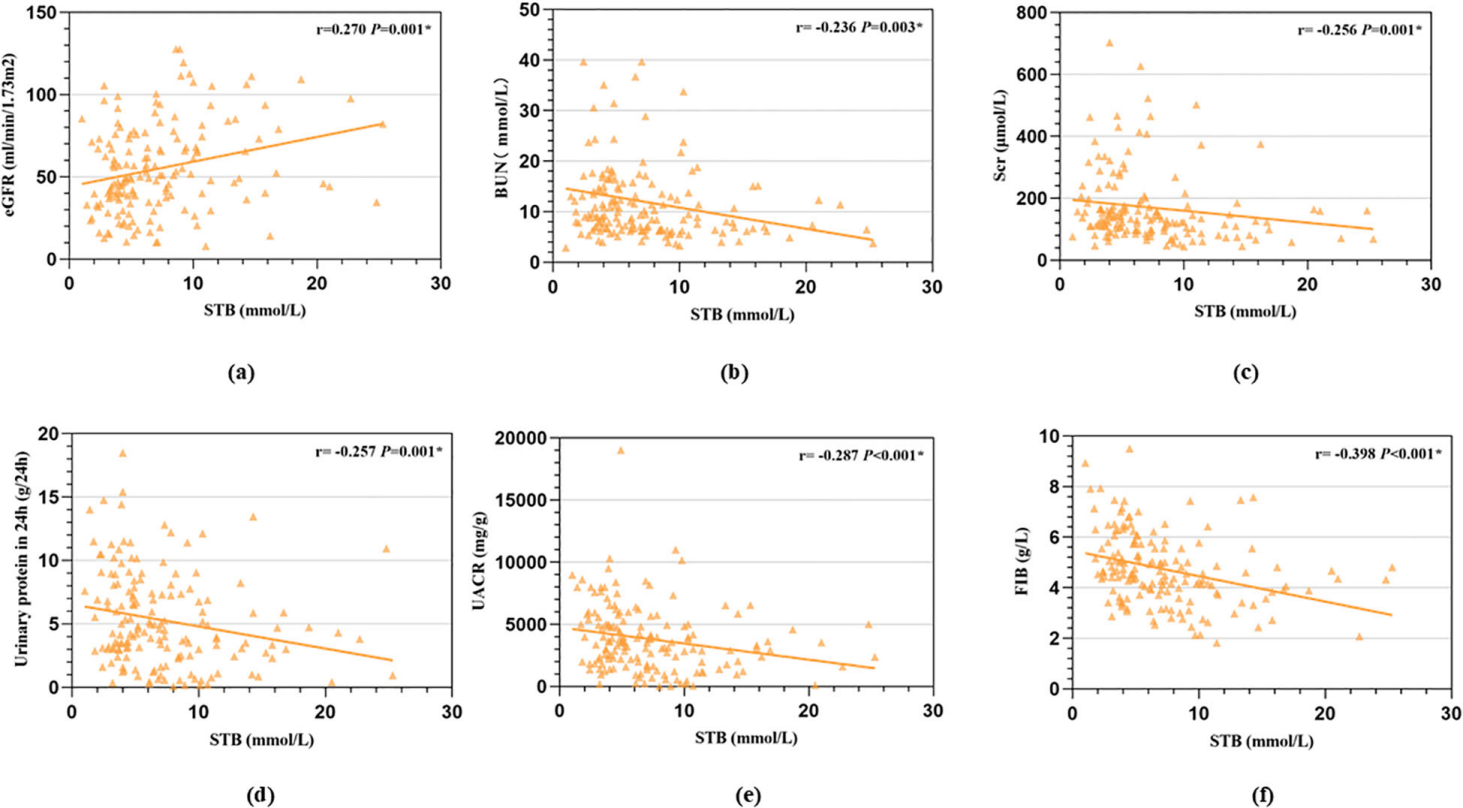
Correlation between STB and renal function indicators calculated by Spearman association analysis. (**a**, STB was positive associated with eGFR; **b**, STB was negative associated with serum BUN; **c**, STB was negative associated with serum creatinine; **d**, STB was negative associated with 24h urine protein; **e**, STB was negative associated with urinary albumin to creatinine ratio; **f**, STB was negative associated with Fib).

Spearman association analysis was used to calculate the association between STB and renal function indicators, we found that STB was positive associated with eGFR (*r* = 0.270, *P* < 0.001, **Figure 1A**) while negative associated with serum BUN (*r* = -0.236, *P* = 0.003, **Figure 1B**), serum creatinine(*r* = -0.256, *P* < 0.001, **Figure 1C**), 24h urine protein(r = -0.257, *P* < 0.001, **Figure 1D**), UACR (*r*=-0.287, *P* < 0.001, **Figure 1E**) and Fib (*r*=-0.398, *P* < 0.001, **Figure 1F**). Patients with higher STB tended to have lower level of BUN, serum creatinine, 24h urine protein, UACR and Fib, but higher level of eGFR.

### 3.3 Survival curve by Kaplan Meier analysis

**Figure 2:**
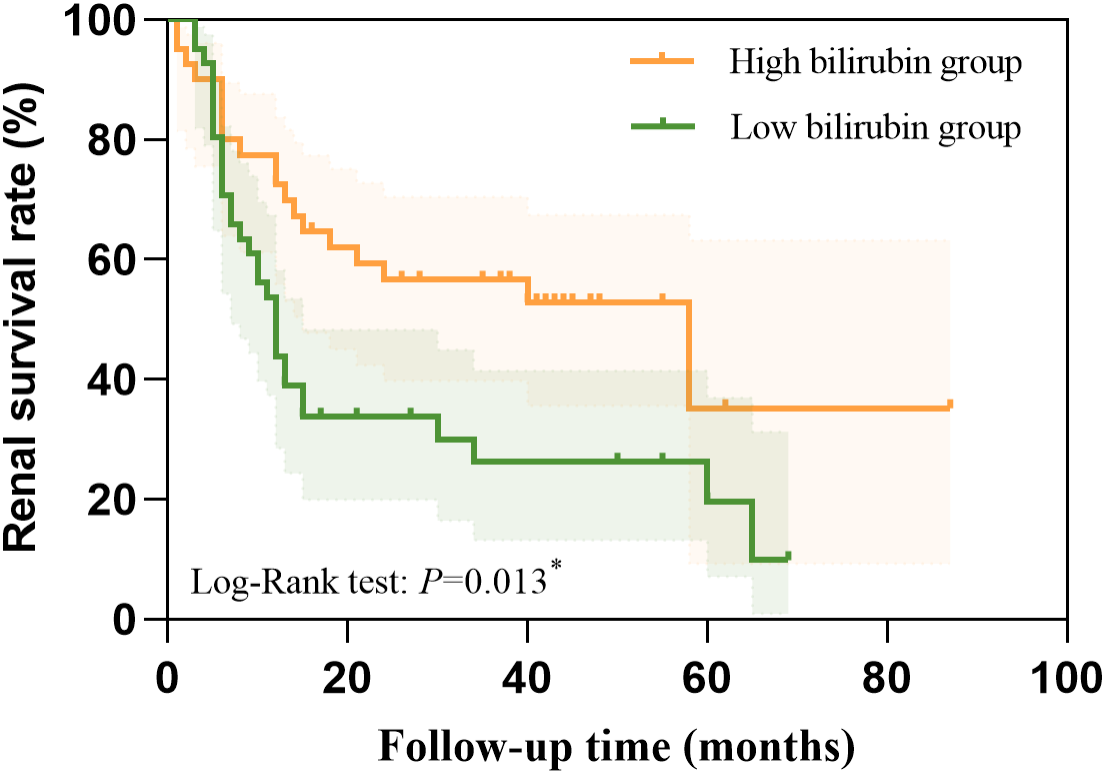
The higher STB had a higher possibility of renal survival rate when compared with lower STB calculated by the Kaplan Meier analysis.

We used Kaplan Meier analysis to performed the survival curve. The Kaplan Meier analysis revealed that high STB had a higher possibility of renal survival rate when compared with lower STB (*P* = 0.013, **Figure 2**).

We also conducted a Cox proportional hazards regression to quantify the effect size. We found STB (*HR*=0.445, 95%*CI*:0.274-0.722, *P*=0.001) were independently protective factors for the DN progression. This indicates that patients in the high bilirubin group have a 0.445-fold lower risk of experiencing a renal event compared to those in the low bilirubin group.

### 3.4 Univariate and multivariate Cox regression analysis

To explore the risk factor of DN progression, we used univariate and multivariate Cox regression analysis. In the univariate regression analysis, we found that age, STB, DBIL, IBIL, eGFR, Scr, eGFR, BUN, 24h Urinary protein, UACR, hemoglobin, PLT, TC, TG, HDL-C, fibrinogen, Serum C4, ACEI/ARB, K-W nodules, renal tubular atrophy and renal interstitial inflammation were risk factors of DN progression (**Table 2**).

**Table 2:** Univariate COX regression analysis of risk factors affecting the progression of DN.

Factors	Univariate	
	HR (95% CI)	*P* value
Clinical characteristics		
Age	0.977(0.959-0.995)	0.014*
Gender	0.908(0.584-1.410)	0.666
DM duration	1.002(0.999,1.004)	0.148
BMI	1.020(0.948,1.097)	0.592
MAP	0.997(0.984, 1.010)	0.661
Hypertension	0.868(0.462,1.633)	0.661
ACEI/ARB	0.224(0.145, 0.348)	<0.001*
STB level	0.418(0.271-0.645)	<0.001*
DBIL	0.740(0.594, 0.923)	0.007
IBIL	0.790(0.706, 0.883)	<0.001*
Scr	1.005(1.004,1.006)	<0.001*
eGFR	0.967(0.957,0.978)	<0.001*
BUN	1.031(1.020,1.042)	<0.001*
Urinary protein in 24h	1.118(1.059,1.181)	<0.001*
UACR	1.000(1.000,1.000)	0.014
HbA1c	1.001(0.965,1.040)	0.942
Serum albumin	0.970(0.941,1.001)	0.055
CRP	1.004(0.986,1.021)	0.694
Hb	0.973(0.963,0.984)	<0.001*
PLT	1.003(1.001,1.005)	0.004*
TC	1.093(1.009,1.183)	0.029*
TG	1.141(1.021,1.276)	0.020*
HDL-C	0.592(0.366,0.958)	0.033*
LDL-C	1.119(0.998,1.254)	0.055
ALT	0.997(0.987, 1.007)	0.562
AST	0.988(0.967, 1.009)	0.243
FIB	1.253(1.093,1.437)	0.001*
Uric acid	1.001(0.999,1.003)	0.481
Urine RBC counts	0.994(0.981,1.006)	0.320
Serum C3	1.490(0.561,3.958)	0.424
Serum C4	6.805(2.225,20.814)	0.001*
Serum C1q	1.001(0.995,1.006)	0.849
Pathological feature		
glomerulosclerosis rate	1.005(0.998,1.011)	0.197
K-W nodules	1.689(1.057,2.697)	0.028*
Renal tubular atrophy (compared with 0 or 1 point)		
2 points	2.292(1.193,4.402)	0.013*
3 points	3.814(2.128,6.837)	<0.001*
4 points	8.555(3.067,23.865)	<0.001*
Renal interstitial inflammation (2 points compared with 0 or 1 point)	2.479(1.602,3.837)	<0.001*
Vascular scores (compared with 0 point)		
1 point	0.486(0.167,1.415)	0.186
2 points	0.483(0.175,1.337)	0.161
3 points	0.693(0.208,2.309)	0.550

Univariate Cox regression model were employed to calculate the hazard ratio (HR) and 95% confidence interval (95% CI) for the association between STB level and DN progression.

^*^ *P* value<0.05.

DN, diabetes nephropathy; STB, serum total bilirubin; DM, diabetes mellitus; BMI, body mass index; MAP, mean arterial pressure; ACEI/ARB, angiotensin-converting enzyme inhibitor/angiotensin receptor blockers; DBIL, direct bilirubin; IBIL, indirect bilirubin; HbA1c, glycosylated hemoglobin, type A1c; TC, total cholesterol; TG, triglycerides; HDL-C, high density lipoprotein cholesterol; LDL-C, low density lipoprotein cholesterol; ALT, alanine transaminase; AST, aspartate transaminase; Scr, serum creatinine; eGFR, estimated glomerular filtration rate; BUN, blood urea nitrogen; UACR, urinary albumin to-creatinine ratio; CRP, C-reactive protein; Hb, hemoglobin; PLT, platelet; FIB, fibrinogen; RBC, red blood cell.

**Figure 3:**
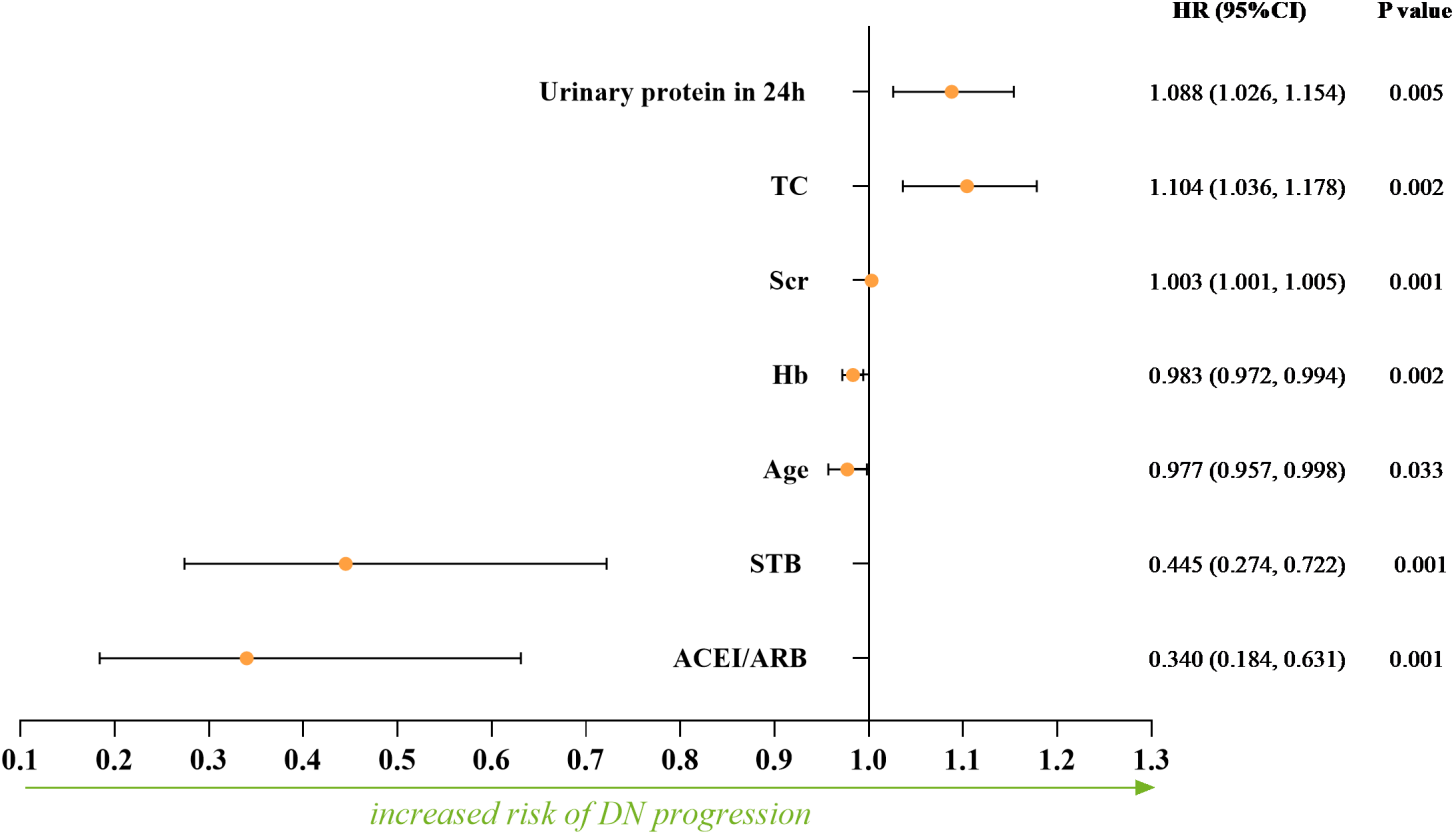
The independent protective factors and risk factors for the DN progression calculated by multivariate Cox regression.

Furtherly, we conducted multivariate Cox regression. Age, STB, DBIL, IBIL, eGFR, Scr, BUN, 24h Urinary protein, UACR, hemoglobin, PLT, TC, TG, HDL-C, fibrinogen, Serum C4, ACEI/ARB, *K-W* nodules, Renal tubular atrophy and Renal interstitial inflammation were included in the adjusted model for the multivariate Cox regression. Finally, STB (*HR* = 0.445, 95%*CI*: 0.274-0.722, *P* = 0.001), hemoglobin (HR = 0.983, 95%*CI*: 0.972-0.994, *P* = 0.002), age (*HR* = 0.977, 95%*CI*: 0.957-0.998, *P* = 0.033) and ACEI/ARB (*HR* = 0.340 95%*CI*: 0.184-0.631, *P* = 0.001) were independently protective factors for the DN progression, while Scr (*HR* = 1.003, 95%*CI*: 1.001-1.005, *P =*0.001), urine protein in 24h (*HR* = 1.088, 95%*CI*: 1.026-1.154, *P* =0.005) and TC (*HR* = 1.104, 95%*CI*: 1.036-1.178, *P =*0.002) were risk factors for the DN progression (**Figure 3**).

### 3.5 The restricted cubic spline model

**Figure 4:**
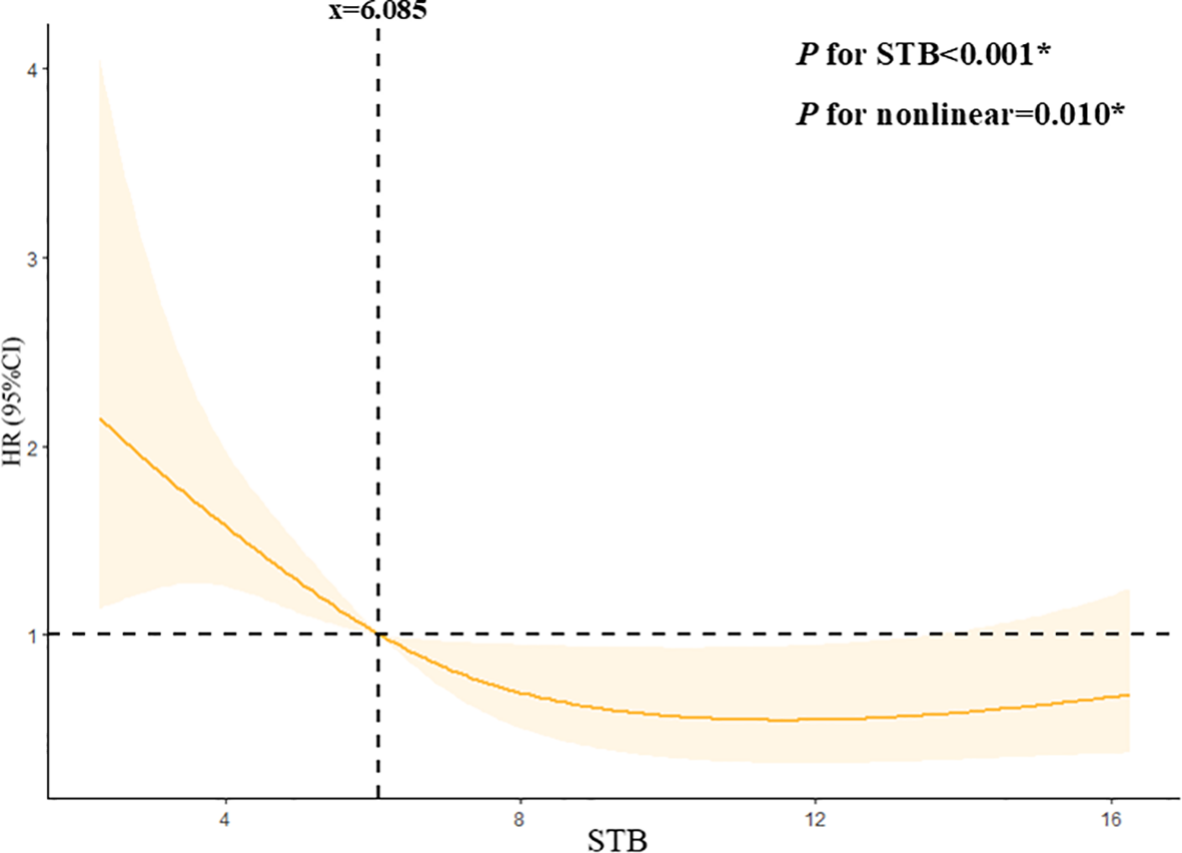
Continuous association of STB with the DN progression by a restricted cubic spline. STB was independently associated with the DN progression when it less than 6.085mmol/L. The restricted cubic spline was plotted using five default knots. The P-value for the nonlinear association was 0.010.

From the restricted cubic spline model, we can see STB was independently associated with DN progression when it was less than 6.085 µmol/L. The restricted cubic spline was plotted using five default knots. The *P*-value for the nonlinear association was 0.010 (**Figure 4**).

## 4 Discussion

In this study, we found that low physiological serum total bilirubin concentrations was a risk factor for disease progression in diabetic nephropathy, which was similar to other studies (13, 14) According to a meta-analysis, there was a negative relationship existing between bilirubin levels and diabetic nephropathy (15). Another 5 years follow-up study found that both serum total bilirubin concentrations and its fluctuation were related to the development of diabetic nephropathy (16). Our study significantly extends prior evidence by providing histopathological validation. Furthermore, we identified a clinically actionable STB threshold (6.085μmol/L) through restricted cubic spline analysis. We elevated STB from a biomarker to a potential therapeutic lever in DN management.

A recent Chinese cohort study demonstrated that maintaining serum bilirubin concentrations within the normal physiological range significantly slows DKD progression, suggesting its hormone-like properties may mediate these renoprotective mechanisms (17). However, large-sample analyses conducted on a US diabetic cohort indicated that the relationship between STB levels and DKD might vary significantly among different populations (18). As such, factors like race, sex, and age need to be carefully considered and relevant inferences should be interpreted cautiously (18). Genetically, Chinese populations possess distinct genetic backgrounds. For instance, variations in genes related to metabolism and inflammation may influence the relationship between serum total bilirubin and diabetic nephropathy. Genetic polymorphisms in pathways regulating bilirubin metabolism, such as the UDP-glucuronosyltransferase 1A1 (UGT1A1) gene, have been shown to differ between Chinese and Western populations (19). These genetic differences can lead to variations in baseline bilirubin levels and its physiological functions, potentially affecting the development and progression of renal damage in diabetic nephropathy.

Our study uniquely advances the understanding of bilirubin’s role in diabetic nephropathy by integrating renal biopsy data, a critical methodological distinction from prior population-based studies. Histopathological analysis revealed that lower serum total bilirubin group specifically associates with higher rate of K-W nodules, renal tubular atrophy and renal interstitial inflammation. Our findings indicate that lower STB levels may compromise the kidney’s antioxidant and ant-inflammatory defenses, thereby promoting the development of these tubulointerstitial lesions. These pathological changes are closely linked to the decline in renal function and disease progression in DN.

Previous studies, mainly using linear models, oversimplified the role of STB in renal function. Our study shows a nonlinear relationship between STB and renal outcomes when STB is below 6.085 µmol/L, indicating that low STB levels affect diabetic nephropathy progression non-linearly. This may be due to the concentration-dependent antioxidant and anti-inflammatory effects of bilirubin, with its protective ability weakening below 6.085 µmol/L and triggering other pathological pathways.

As we known, senescent or damaged erythrocytes decompose and liberate heme. Heme oxygenase mediates the catabolism of heme, resulting in the production of carbon monoxide and biliverdin. Biliverdin reductase further reduces biliverdin to bilirubin, which allows cells to avoid heme accumulation. *In vitro* and *in vivo* experiments have confirmed that bilirubin has powerful anti-inflammatory and antioxidant properties. Given that the development of diabetic nephropathy is associated with inflammation and oxidative stress, a similar relationship has been studied between bilirubin levels and ischemic stroke, coronary heart disease, and atherosclerosis (19). These clinical findings appear to be related to the role of bilirubin in immunosuppression and inhibition of protein phosphorylation (20). In *in vitro* and *in vivo* experiments, the immunomodulatory effects of bilirubin are reflected in the fact that mild elevations in bilirubin concentrations attenuate endoplasmic reticulum stress and reduce levels of inflammatory cytokines (21).

The current protective mechanism of bilirubin against diabetic nephropathy focuses on both antioxidant and anti-inflammatory pathways. Kumar (22) found that serum bilirubin level was negatively correlated with the level of oxidative stress, and positively correlated with the level of antioxidant enzymes such as superoxide dismutase, catalase, and glutathione peroxidase. Bilirubin improves endothelium-dependent dilatation in the aorta of diabetic mice through the protein kinase B/endothelial nitric oxide synthase/nitric oxide (Akt/eNOS/NO) cascade (23). Hyperglycemia in diabetes leads to the overproduction of reactive oxygen species (ROS) in renal cells, including glomerular endothelial cells, podocytes, and tubular epithelial cells. ROS can damage cellular components such as DNA, proteins, and lipids, and also trigger the activation of pro-inflammatory pathways. The more severe pathological manifestations in the low bilirubin group may be partly attributed to insufficient antioxidant protection. Bilirubin’s antioxidant properties could potentially play a crucial role in mitigating this oxidative stress in DN. By scavenging free radicals, bilirubin may protect renal cells from ROS-induced damage, thereby preserving the integrity of the glomerular filtration barrier and reducing the progression of proteinuria. For example, bilirubin can directly react with superoxide anions and hydroxyl radicals, two highly reactive and damaging ROS species, to prevent them from causing cellular injury (24).

The anti-inflammatory potential manifests through multifaceted interactions: suppression of bacterial endotoxin-induced inflammation, inhibition of vascular adhesion molecule expression, attenuation of glomerulosclerosis, and reduction of pro-inflammatory cytokine cascades. Notably, bilirubin exhibits targeted modulation of renal inflammatory infiltrates and vascular remodeling processes characteristic of diabetic nephropathy progression. The protective role of bilirubin against DN progression, as evidenced by its ability to mitigate podocyte apoptosis and oxidative stress through enhanced cellular defense pathways in hyperbilirubinemic models (25), supports our observation linking reduced physiological bilirubin levels to accelerated renal injury. Bilirubin may modulate the activation of immune cells and the production of pro-inflammatory cytokines in the kidneys. In DN, the infiltration of immune cells such as macrophages and T-lymphocytes into the renal interstitium contributes to the inflammatory process. The increased renal interstitial inflammation in the low bilirubin group may be related to a lack of bilirubin-mediated anti-inflammatory regulation. A study on type 2 diabetic rats showed that bilirubin treatment improved glomerular structural damage, reversed the reduction in kidney and related structure volumes, indicating bilirubin’s potential protective effects on diabetic kidney disease progression by alleviating inflammation (26).

This study also has some limitations. Firstly, it was a single-center retrospective study, the sample size is limited, suggesting that there might have been selection bias. Further studies with larger sample sizes and follow-up are needed to determine the contribution of serum total bilirubin levels to the disease progression of diabetic nephropathy. Secondly,the lack of data on antidiabetic treatments limits the understanding of the complex factors influencing diabetic nephropathy progression. In future research, it will be essential to incorporate this variable for a more in-depth analysis. Finally, single-timepoint STB measurement may not capture longitudinal variations. Future prospective studies should incorporate serial bilirubin assessments to evaluate its dynamic relationship with DKD progression. What was more, using RRT initiation as the criterion for progression may underdetect early DN progression signals. Future studies should integrate STB with serial biomarker and histopathological assessments to capture earlier progression phases.

## 5 Conclusions

In summary, our findings suggest that STB may serve as a potential biomarker for the progression of diabetic nephropathy because of its reduced anti-inflammatory and vasoprotective effects. Lower STB levels may help identify high-risk patients who could benefit from earlier or more intensive interventions to slow disease progression.

## Data Availability

The original contributions presented in the study are included in the article/**Supplementary Material**. Further inquiries can be directed to the corresponding authors.
