# Supplementary material for: Association between physiological serum total bilirubin concentrations and the progression of diabetic nephropathy
Source: Front Endocrinol (Lausanne). 2025 May 29;16:1588568. doi: 10.3389/fendo.2025.1588568 (PMC12158686; doi:10.3389/fendo.2025.1588568)
Supplement: Supplementary file 1 [file Table1.docx]

**Supplement Table1** Comparison of clinicopathological data between low bilirubin and high bilirubin groups of eGFR＜60ml/min/1.73m^2^ and ≥60ml/min/1.73m^2^ DN patients.

|  | **eGFR＜60ml/min/1.73m2** | | | |  | **eGFR≥60ml/min/1.73m2** | | | |
| --- | --- | --- | --- | --- | --- | --- | --- | --- | --- |
|  | overall (N=98) | Low bilirubin group (N=58) | High bilirubin group (N=40) | *P*-value |  | overall (N=61) | Low bilirubin group (N=22) | High bilirubin group (N=39) | *P* value |
| **Clinical characteristics** | |  |  |  |  |  |  |  |  |
| Age (years) | 56.00(48.00,62.00) | 55.50(48.00,62.00) | 56.00(48.00,61.75) | 0.965 |  | 52.61±11.43 | 52.36±11.72 | 52.74±11.41 | 0.902 |
| Gender  (male,N,%) | 67(68.40%) | 36(62.10%) | 31(77.50%) | 0.106 |  | 41(67.20%) | 16(72.70%) | 25(64.10%) | 0.491 |
| DM duration (months) | 84.00  (36.00,144.00) | 96.00  (36.00,156.00) | 72.00  (21.00,120.00) | 0.264 |  | 72.00  (30.00, 120.00) | 72.00  (36.00, 120.00) | 72.00  (24.00, 120.00 | 0.862 |
| BMI (kg/m2) | 23.89±2.88 | 23.84±3.07 | 23.96±2.63 | 0.826 |  | 23.74±3.08 | 23.92±3.46 | 23.64±2.90 | 0.737 |
| MAP (mmHg) | 104.54±15.89 | 102.90±13.66 | 106.93±18.59 | 0.219 |  | 104.90±14.92 | 106.27±17.14 | 104.13±13.69 | 0.594 |
| STB(µmol/L) | 5.05(3.88,7.80) | 4.00(3.28, 4.73) | 8.30(7.03, 11.40) | <0.001* |  | 7.40(4.45, 10.50) | 3.80(2.80, 4.83) | 10.00(8.40, 13.40) | <0.001* |
| DBIL (µmol/L) | 1.90(1.18, 2.06) | 1.40(1.00, 2.04) | 2.04(1.93, 2.63) | <0.001* |  | 2.04(1.30, 2.95) | 1.15(0.70, 1.63) | 2.60(2.04, 3.50) | <0.001* |
| IBIL (µmol/L) | 3.70(2.38, 4.23) | 3.15(1.78, 4.21) | 4.23(3.43, 5.88) | <0.001* |  | 4.40(3.15, 6.15) | 2.75(2.13, 3.93) | 5.90(4.40, 8.10) | <0.001* |
| ACEI/ARB (N,%) | 59(60.2 %) | 36(62.1 %） | 23(57.50%) | 0.650 |  | 59(96.70%) | 21(95.50%) | 38(97.40%) | 0.676 |
| Scr (µmol/L) | 160.95  (132.90,269.68) | 162.50  (132.90,275.88) | 159.00  (130.00,255.30) | 0.942 |  | 86.00  (69.90, 108.90) | 96.30  (79.68, 111.60) | 80.00  (65.00, 99.00) | 0.035* |
| eGFR (ml/min/1.73m^2^) | 39.86(27.25, 48.06) | 39.22(26.12, 46.52) | 40.83(29.25,50.56) | 0.448 |  | 79.08(67.82, 96.04) | 75.84(67.14, 83.41) | 84.03  (67.99, 105.28) | 0.058 |
| BUN（mmol/L） | 12.06(8.01,16.58) | 12.12(8.77,16.58) | 11.49(7.20,16.71) | 0.485 |  | 6.51(5.65, 9.12) | 7.89(6.09, 9.87) | 6.37(5.46, 8.44) | 0.099 |
| Urinary protein in 24h (g/24h) | 4.76(2.99,8.99) | 5.39(3.10,10.19) | 4.40(1.78,7.34) | 0.081 |  | 3.75(2.43, 6.37) | 5.34(3.19, 7.18) | 3.34(1.10, 5.25) | 0.026* |
| UACR (mg/g) | 3771.34  (2112.64,5614.48) | 4000.00  (2340.66,6039.31) | 3088.15  (1427.04,4237.50) | 0.021* |  | 3000.00  (1494.81, 4776.37) | 3442.78  (2429.26, 6000.00) | 2675.99  (1200.00, 3977.00) | 0.020* |
| HbA1c (%) | 7.25(6.18,8.90) | 7.45(6.40,9.03) | 7.05(6.00,8.58) | 0.261 |  | 8.30(7.05, 9.80) | 7.95(6.78, 9.75) | 8.40(7.10, 10.40) | 0.641 |
| Serum albumin (g/L) | 29.05(26.88, 34.73) | 28.70(26.55, 34.45) | 29.95(27.10, 34.88) | 0.441 |  | 30.90(24.25, 35.10) | 27.35(23.98, 34.53) | 31.60(24.40, 36.50) | 0.453 |
| Hb（g/L） | 102.57±20.72 | 98.71±17.16 | 108.18±24.14 | 0.025* |  | 115.28±23.79 | 110.50±19.07 | 117.97±25.92 | 0.204 |
| PLT (10^9/L) | 245.50  (202.25,306.50) | 254.50  (210.00,318.25) | 241.00  (190.75,278.00) | 0.080 |  | 233.00  (194.50, 312.00) | 274.50  (209.75, 369.75) | 231.00  (185.00, 284.00) | 0.051 |
| FIB (g/L) | 4.91±1.43 | 5.28±1.44 | 4.37±1.26 | 0.002* |  | 4.28(3.41, 5.08) | 5.08(4.30, 5.82) | 4.13(3.10, 4.81) | <0.001* |
| Uric acid  (µmol/L) | 392.60  (333.75, 442.55) | 395.40  (336.50, 434.08) | 385.55  (328.85, 463.93) | 0.800 |  | 371.98±87.61 | 346.55±70.06 | 386.33±93.92 | 0.089 |
| TC (mmol/L) | 5.06(3.71, 5.27) | 5.60(4.04, 6.56) | 4.58(3.43, 6.00) | 0.126 |  | 5.70±1.97 | 5.96±1.80 | 5.55±2.08 | 0.442 |
| TG (mmol/L) | 1.63(1.09, 2.30) | 1.80(1.18, 3.41) | 1.57(1.02, 1.83) | 0.043* |  | 1.75(1.20, 2.40) | 1.67(1.23, 2.55) | 1.76(1.07, 2.36) | 0.851 |
| HDL-C (mmol/L) | 1.07±0.38 | 1.05±0.36 | 1.10±0.40 | 0.501 |  | 1.14(0.98, 1.42） | 1.10(0.94, 1.40) | 1.15(1.00, 1.43) | 0.457 |
| LDL-C (mmol/L) | 3.13(2.16, 4.32) | 3.16(2.04, 4.36) | 2.90(2.18, 4.29) | 0.859 |  | 3.67±1.41 | 3.89±1.45 | 3.54±1.39 | 0.363 |
| ALT (U/L) | 20.00(13.00, 23.00) | 17.00(12.00, 23.00) | 23.00(15.50, 23.00) | 0.060 |  | 16.00(11.50, 23.00) | 14.50(12.50, 23.00) | 16.00(11.00, 23.00) | 0.646 |
| AST (U/L) | 20.00(15.75, 23.00) | 18.50(15.00, 23.00) | 22.00(17.25, 22.00) | 0.228 |  | 19.00(14.00, 25.00) | 20.00(13.00, 25.00) | 19.00(16.00, 26.00) | 0.690 |
| Serum C3 (g/L) | 0.88(0.77, 0.98) | 0.81(0.89,1.03) | 0.87(0.78,0.90) | 0.010* |  | 0.89(0.82, 1.04) | 0.89(0.83, 1.10) | 0.89(0.82, 1.02) | 0.421 |
| Progression (N,%) | 70(71.40%) | 45(77.60%) | 25(62.50%） | 0.104 |  | 20(32.80%) | 12(54.50%) | 8(20.50%) | 0.007* |

**Table2 (continued)**

|  | **eGFR＜60ml/min/1.73m2** | | | |  | **eGFR≥60ml/min/1.73m2** | | | |
| --- | --- | --- | --- | --- | --- | --- | --- | --- | --- |
|  | overall (N=98) | Low bilirubin group (N=58) | High bilirubin group (N=40) | *P*-value |  | overall (N=61) | Low bilirubin group (N=22) | High bilirubin group (N=39) | *P* value |
| **Pathological feature** | |  |  |  |  |  |  |  |  |
| Globular glomerulosclerosis rate (%) | 24.55(9.10,40.35) | 25.00(9.78,42.83) | 21.15(8.50,39.10) | 0.331 |  | 18.80(7.10, 33.30) | 23.20(5.33, 40.60) | 18.20(7.10, 33.30) | 0.587 |
| Segmental glomerulosclerosis rate (%) | 7.70(0.00,37.55) | 7.20(0.00,34.08) | 9.35(0.00,42.60) | 0.715 |  | 0.00(0.00, 27.95) | 15.50(0.00, 34.98) | 0.00(0.00, 18.40) | 0.263 |
| K-W nodules (N,%) | 65(66.30%) | 41(70.70%) | 24(60.00%) | 0.271 |  | 38(62.30%) | 19(86.40%) | 19(48.70%) | 0.004* |
| Renal tubular atrophy(0/1/2/3/4) | 1/15/28/49/5 | 0/7/17/31/3 | 1/8/11/18/2 | 0.594 |  | 0/32/17/12/0 | 0/8/6/8/0 | 0/24/11/4/0 | 0.037* |
| Renal interstitial inflammation (0/1/2) | 1/35/62 | 0/19/39 | 1/16/23 | 0.341 |  | 1/41/19 | 0/12/10 | 1/29/9 | 0.161 |
| Vascular scores (0/1/2/3) | 4/30/54/10 | 3/13/37/5 | 1/17/17/5 | 0.120 |  | 2/13/46/0 | 0/2/20/0 | 2/11/26/0 | 0.098 |
| Data are expressed as means±standard deviation or medians (interquartile range) or count (%).  ^*^*P* value<0.05.  Abbreviations: DN: diabetes nephropathy; STB: serum total bilirubin; DM: diabetes mellitus; BMI: body mass index; HbA1c: glycosylated hemoglobin, type A1c; Scr: serum creatinine; eGFR: estimated glomerular filtration rate; Hb: hemoglobin; PLT: platelet; BUN: blood urea nitrogen; UACR: urinary albumin to-creatinine ratio; FIB: fibrinogen; MAP: mean arterial pressure; ACEI/ARB: angiotensin-converting enzyme inhibitor/angiotensin receptor blockers; DBIL: direct bilirubin; IBIL: indirect bilirubin; ALT: alanine transaminase; AST: aspartate transaminase. | | | | | | | | | |
