# Supplementary material for: Association between physiological serum total bilirubin concentrations and the progression of diabetic nephropathy
Source: Front Endocrinol (Lausanne). 2025 May 29;16:1588568. doi: 10.3389/fendo.2025.1588568 (PMC12158686; doi:10.3389/fendo.2025.1588568)
Supplement: Supplementary file 2 [file Table2.docx]

**Supplement Table2** Univariate COX regression analysis of factors affecting the development of DN in eGFR＜60ml/min/1.73m^2^ and ≥60ml/min/1.73m^2^ groups

| Factors | **eGFR＜60ml/min/1.73m2** | |  | **eGFR≥60ml/min/1.73m2** | |
| --- | --- | --- | --- | --- | --- |
|  | HR (95% CI) | *P* value |  | HR (95% CI) | *P* value |
| **Clinical characteristics** |  |  |  |  |  |
| Age | 0.963(0.941, 0.984) | 0.001* |  | 0.987(0.949, 1.025) | 0.492 |
| Gender | 0.722(0.440, 1.188) | 0.200 |  | 1.366(0.513, 3.639) | 0.533 |
| DM duration | 1.001(0.999, 1.004) | 0.278 |  | 1.001(0.995, 1.007) | 0.829 |
| BMI | 1.032(0.941, 1.132) | 0.500 |  | 1.001(0.869, 1.153) | 0.989 |
| MAP | 1.000(0.985, 1.014) | 0.958 |  | 0.989(0.960, 1.019) | 0.479 |
| Hypertension | 0.778(0.397, 1.523) | 0.463 |  | 2.177(0.291, 16.276) | 0.448 |
| ACEI/ARB | 0.324(0.200, 0.525) | <0.001* |  | 0.207(0.026, 1.647) | 0.137 |
| STB level | 0.584(0.356, 0.959) | 0.034* |  | 0.312(0.1127, 0.765) | 0.011* |
| DBIL | 0.782(0.597, 1.025) | 0.075 |  | 0.709(0.461, 1.090) | 0.117 |
| IBIL | 0.868(0.762, 0.990) | 0.035* |  | 0.735(0.581, 0.931) | 0.011* |
| Scr | 1.004(1.002, 1.005) | <0.001* |  | 1.018(1.009, 1.028) | <0.001* |
| eGFR | 0.955(0.938, 0.972) | <0.001* |  | 0.989(0.963, 1.015) | 0.399 |
| BUN | 1.069(1.041, 1.099) | <0.001* |  | 1.040(1.016, 1.066) | 0.001* |
| Urinary protein in 24h | 1.060(0.998, 1.126) | 0.058 |  | 1.213(1.067, 1.378) | 0.003* |
| UACR | 1.000(1.000, 1.000) | 0.544 |  | 1.000(1.000, 1.000) | 0.002 |
| HbA1c | 1.000(0.968, 1.034) | 0.995 |  | 1.072(0.898, 1.280) | 0.443 |
| Serum albumin | 0.967(0.931, 1.005) | 0.085 |  | 0.957(0.899, 1.018) | 0.165 |
| CRP | 0.992(0.970, 1.014) | 0.475 |  | 1.026(0.994, 1.060) | 0.107 |
| Hb | 0.980(0.968, 0.992) | 0.002* |  | 0.967(0.946, 0.989) | 0.003* |
| PLT | 1.003(1.001, 1.006) | 0.016* |  | 1.004(1.000, 1.008) | 0.076 |
| TC | 1.127(1.050, 1.209) | 0.001* |  | 1.001(0.807, 1.242) | 0.990 |
| TG | 1.118(0.991, 1.261) | 0.069 |  | 1.215(0.951, 1.552) | 0.120 |
| HDL-C | 0.878(0.466, 1.654) | 0.688 |  | 0.468(0.172, 1.274) | 0.137 |
| LDL-C | 1.134(1.013, 1.268) | 0.028* |  | 1.177(0.883, 1.570) | 0.266 |
| ALT | 0.996(0.987, 1.006) | 0.465 |  | 0.973(0.929, 1.019) | 0.246 |
| AST | 0.991(0.968, 1.014) | 0.424 |  | 0.970(0.921, 1.022) | 0.257 |
| FIB | 1.123(0.958, 1.315) | 0.152 |  | 1.543(1.1157, 2.057) | 0.003* |
| Uric acid | 0.999(0.997, 1.002) | 0.598 |  | 1.003(0.998, 1.008) | 0.228 |
| Urine RBC counts | 0.998(0.985, 1.011) | 0.798 |  | 0.970(0.925, 1.017) | 0.208 |
| Serum C3 | 1.424(0.447, 4.541) | 0.550 |  | 4.810(0.711, 32.555) | 0.107 |
| **Pathological feature** |  |  |  |  |  |
| glomerulosclerosis rate | 1.004(0.997, 1.012) | 0.280 |  | 0.996(0.981, 1.012) | 0.640 |
| K-W nodules | 1.422(0.844, 2.395) | 0.186 |  | 2.747(0.917, 8.232) | 0.071 |

**Table4 (continued)**

| Factors | **eGFR＜60ml/min/1.73m2** | |  | **eGFR≥60ml/min/1.73m2** | |
| --- | --- | --- | --- | --- | --- |
|  | HR (95% CI) | *P* value |  | HR (95% CI) | *P* value |
| Renal tubular atrophy  (compared with 0 or 1 point) |  |  |  |  |  |
| 2 points | 2.618(1.035, 6.625) | 0.042* |  | 1.263(0.423, 3.774) | 0.676 |
| 3 points | 3.595(1.521, 8.498) | 0.004* |  | 0.961(0.697, 5.518) | 0.202 |
| 4 points | 6.470(1.947, 21.498) | 0.002* |  | - | - |
| Renal interstitial inflammation  (2 points compared with 0 or 1 point) | 1.824(1.083, 3.074) | 0.024* |  | 2.103(0.870, 5.086) | 0.099 |
| Vascular scores  (compared with 0 or 1 point) |  |  |  |  |  |
| 2 points | 1.056(0.630, 1.771) | 0.836 |  | 1.469(0.426, 5.058) | 0.543 |
| 3 points | 0.911(0.405, 2.052) | 0.823 |  | - | - |
| Univariate Cox regression model were employed to calculate the hazard ratio (HR) and 95% confidence interval (95% CI) for the association between STB level and DN progression.  ^*^*P* value<0.05.  Abbreviations: DN: diabetes nephropathy; STB: serum total bilirubin; DM: diabetes mellitus; BMI: body mass index; MAP: mean arterial pressure; ACEI/ARB: angiotensin-converting enzyme inhibitor/angiotensin receptor blockers; DBIL: direct bilirubin; IBIL: indirect bilirubin; HbA1c: glycosylated hemoglobin, type A1c; TC: total cholesterol; TG: triglycerides; HDL-C: high density lipoprotein cholesterol; LDL-C: low density lipoprotein cholesterol; ALT: alanine transaminase; AST: aspartate transaminase; Scr: serum creatinine; eGFR: estimated glomerular filtration rate; BUN: blood urea nitrogen; UACR: urinary albumin to-creatinine ratio; CRP: C-reactive protein; Hb: hemoglobin; PLT: platelet; FIB: fibrinogen; RBC: red blood cell. | | | | | |
