# Supplementary material for: Association between physiological serum total bilirubin concentrations and the progression of diabetic nephropathy
Source: Front Endocrinol (Lausanne). 2025 May 29;16:1588568. doi: 10.3389/fendo.2025.1588568 (PMC12158686; doi:10.3389/fendo.2025.1588568)
Supplement: Supplementary file 3 [file Table3.docx]

**Supplement Table3.** Multivariate COX regression analysis of factors affecting the development of DN in eGFR＜60ml/min/1.73m^2^ and ≥60ml/min/1.73m^2^ groups.

| **eGFR**＜60ml/min/1.73m2 | | |  | **eGFR**≥60ml/min/1.73m2 | | |
| --- | --- | --- | --- | --- | --- | --- |
| Factors | *HR* (95% *CI*) | *P* value |  | Factors | *HR* (95% *CI*) | *P* value |
| **STB** | 0.367(0.211, 0.638) | 0.001 |  | **Hb** | 0.975(0.951,0.998) | 0.043 |
| **Scr** | 1.003(1.001, 1.005) | 0.002 |  | **Scr** | 1.012(1.002, 1.023) | 0.018 |
| **TC** | 1.141(1.073, 1.214) | 0.001 |  | **FIB** | 1.508(1.090, 2.087) | 0.013 |
| **ACEI/ARB** | 0.360(0.187, 0.693) | 0.002 |  |  | 1.001(0.869, 1.153) | 0.989 |
