# Supplementary material for: Association between physiological serum total bilirubin concentrations and the progression of diabetic nephropathy
Source: Front Endocrinol (Lausanne). 2025 May 29;16:1588568. doi: 10.3389/fendo.2025.1588568 (PMC12158686; doi:10.3389/fendo.2025.1588568)
Supplement: Supplementary file 4 [file Table4.docx]

**Supplement Table4.** Normality test results for all variables.

|  | | Shapiro-Wilk Test | | |
| --- | --- | --- | --- | --- |
| Variables | Statistic | | Degrees of Freedom | Significance |
| Serum total bilirubin | 0.862 | | 159 | 0.000 |
| Age | 0.98 | | 159 | 0.019 |
| eGFR | 0.969 | | 159 | 0.001 |
| BUN | 0.591 | | 159 | 0.000 |
| Scr | 0.732 | | 159 | 0.000 |
| BMI | 0.989 | | 159 | **0.233** |
| DM duration | 0.899 | | 159 | 0.000 |
| HGB | 0.992 | | 159 | **0.518** |
| PLT | 0.947 | | 159 | 0.000 |
| HbA1c | 0.371 | | 159 | 0.000 |
| Uric acid | 0.967 | | 159 | 0.001 |
| Alb | 0.971 | | 159 | 0.002 |
| TC | 0.726 | | 159 | 0.000 |
| TG | 0.791 | | 159 | 0.000 |
| HDL | 0.856 | | 159 | 0.000 |
| LDL | 0.852 | | 159 | 0.000 |
| Calcium | 0.992 | | 159 | **0.514** |
| Phosphorus | 0.982 | | 159 | 0.037 |
| CRP | 0.546 | | 159 | 0.000 |
| Fib | 0.977 | | 159 | 0.01 |
| C1q | 0.941 | | 159 | 0.000 |
| C3 | 0.921 | | 159 | 0.000 |
| C4 | 0.638 | | 159 | 0.000 |
| Urinary protein in 24h | 0.938 | | 159 | 0.000 |
| ACR | 0.902 | | 159 | 0.000 |
| Urine RBC counts | 0.511 | | 159 | 0.000 |
| MAP | 0.99 | | 159 | **0.332** |
| DBIL | 0.79 | | 159 | 0.000 |
| IBIL | 0.894 | | 159 | 0.000 |
| ALT | 0.465 | | 159 | 0.000 |
| AST | 0.828 | | 159 | 0.000 |
