# Supplementary material for: Association between physiological serum total bilirubin concentrations and the progression of diabetic nephropathy
Source: Front Endocrinol (Lausanne). 2025 May 29;16:1588568. doi: 10.3389/fendo.2025.1588568 (PMC12158686; doi:10.3389/fendo.2025.1588568)
Supplement: Supplementary file 5 [file Table5.docx]

***Supplementary table: detailing for all biochemical parameters.***

|  | **normal reference ranges** | **detection methods** |
| --- | --- | --- |
| **TBil (µmol/L)** | 0-26.0 | Vanadate oxidation method |
| **DBil (µmol/L)** | 0-6.8 | Vanadate oxidation method |
| **IBil (µmol/L)** | 0-19 | calculate |
| **ALT(U/L)** | 9-50 | Lactate Dehydrogenase Method |
| **AST(U/L)** | 15-40 | Malate Dehydrogenase Method |
| **Scr (µmol/L)** | 57-111（male）  41-73（female） | enzymatic assay |
| **eGFR (ml/min/1.73m^2^)** | ＞80 | Calculated by CKD-EPI formula |
| **BUN (mmol/L)** | 3.6-9.5 | Urease method |
| **Urinary protein in 24h (g/24h)** | 0-0.15 | Turbidimetry method and calculate |
| **UACR (mg/g)** | 0-30 | calculate |
| **HbA1c (%)** | 3.9-6.0 | HPLC |
| **Serum albumin (g/L)** | 40.0-55.0 | Bromocresol green method |
| **CRP (mg/L）** | 0-6.0 | Immunoturbidimetry method |
| **Hb (g/L）** | 135-175(male)  115-150(female) | Detected by the BC-6800Plus hematology analyzer |
| **PLT (10^9/L)** | 125-350 | Detected by the BC-6800Plus hematology analyzer |
| **TC (mmol/L)** | ＜5.18 | Oxidase method |
| **TG (mmol/L)** | 0.34-1.70 | Oxidase method |
| **HDL-C (mmol/L)** | ＞1.04 | Selective masking method |
| **LDL-C (mmol/L)** | ＜3.40 | Direct determination method |
| **Calcium (mmol/L)** | 2.11-2.52 | Arsenazo III |
| **Phosphorus (mmol/L)** | 0.85-1.51 | Direct ultraviolet method |
| **FIB (g/L)** | 1.8-3.5 | Clotting method |
| **Uric acid (µmol/L)** | 208-428(male)  155-357(female) | Oxidase method |
| **Serum C3 (g/L)** | 0.76-1.70 | Immunoturbidimetry method |
| **Serum C4 (g/L)** | 0.11-0.32 | Immunoturbidimetry method |
